# Use of Entropy in Developing SDG-based Indices for Assessing Regional Sustainable Development: A Provincial Case Study of China

**DOI:** 10.3390/e22040406

**Published:** 2020-04-02

**Authors:** Xiangyu Wang, Peichao Gao, Changqing Song, Changxiu Cheng

**Affiliations:** 1 State Key Laboratory of Earth Surface Processes and Resource Ecology, Beijing Normal University, Beijing 100875, China; 201831051025@mail.bnu.edu.cn (X.W.); gaopc@bnu.edu.cn (P.G.); 2 Key Laboratory of Environmental Change and Natural Disaster, Beijing Normal University, Beijing 100875, China; 3 Center for Geodata and Analysis, Faculty of Geographical Science, Beijing Normal University, Beijing 100875, China; chengcx@bnu.edu.cn

**Keywords:** sustainable development, index, indicator, SDGs, entropy, Fujian province

## Abstract

Sustainable development appears to be the theme of our time. To assess the progress of sustainable development, a simple but comprehensive index is of great use. To this end, a multivariate index of sustainable development was developed in this study based on indicators of the United Nations Sustainable Development Goals (SDGs). To demonstrate the usability of this developed index, we applied it to Fujian Province, China. According to the China SDGs indicators and the Fujian situation, we divided the SDGs into three dimensions and selected indicators based on these dimensions. We calculated the weights and two indices with the entropy weight coefficient method based on collecting and processing of data from 2007 to 2017. We assessed and analyzed the sustainable development of Fujian with two indices and we drew three main conclusions. From 2007 to 2017, the development index of Fujian showed an increasing trend and the coordination index of Fujian showed a fluctuating trend. It is difficult to smoothly improve the coordination index of Fujian because the development speeds of Goal 3 (Good Health and Well-being) and Goal 16 (Peace, Justice, and Strong Institutions) were low. The coordination index of Fujian changed from strong coordination to medium coordination from 2011 to 2012 because the development speed of the environmental dimension suddenly improved. It changed from strong coordination to medium coordination from 2015 to 2016 because the values of the development index of the social dimension were decreasing. To the best of our knowledge, these are the first SDGs-based multivariate indices of sustainable development for a region of China. These indices are applicable to different regions.

## 1. Introduction

Since the Reform and Opening-up, China has experienced rapid development for approximately forty years. The Chinese people have had great achievements. China’s urbanization increased from 17.92% in 1978 to 59.58% in 2018 [1]. Simultaneously, with the development of industrialization and globalization, China has been suffering from problems such as deforestation, urban expansion, biodiversity conservation, and environmental pollution [2,3,4,5]. To solve these problems, people developed the concept of sustainable development [6,7]. Gradually, the concept of sustainable development has become widely agreed upon by stakeholders and scholars [8]. Stakeholders take measures to implement development under the guidance of the concept of sustainable development.

During the development of a nation or region, stakeholders and scholars need to assess the degree of sustainable development. To this end, one popular method is to construct a composite index based on a number of meaningful indicators. For example, to assess sustainable livelihood, Donohue and Biggs [9] modified the multidimensional livelihood index by selecting 23 indicators. To assess social sustainability, the Sustainable Society Foundation proposed a regional sustainable society index with 21 indicators [10]. Nhemachena, et al. [11] developed a composite baseline index for agriculture by selecting 13 indicators. Costanza, et al. [12] measured wellbeing in connection with the Sustainable Development Goals. There are other indices, namely, the FEEM sustainability index, which includes 19 indicators [13], and the index of essential health services, which includes 16 indicators [14]. Indicators form the basis of a composite index. However, we argue that more integration is needed between the composite index for assessing regional sustainable development and the indicators of Sustainable Development Goals (SDGs).

Therefore, in the present study, we aimed to evaluate SDG-based indices for assessing regional sustainable development. The SDGs were launched by the United Nations in 2015 [15], containing a total of 17 goals, 169 targets, and 232 indicators. This constructs a development blueprint for the future, such as for education, equality, biodiversity, hunger, and pollution. Scholars believe that the SDG indicators are the most comprehensive for assessing sustainable development [16,17]. There are two dominant methods for assessing sustainable development based on SDGs. First, the “SDG Index and Dashboards Report”, prepared by the Bertelsmann Stiftung and Sustainable Development Solutions Network (SDSN) since 2016 [18], developed a method for constructing the SDG Index. The core of this method bears equal weight to every SDG and has a lot of applications at different scales. Specific editions exist at continental, national, and regional levels, such as Africa [19], the European Union [20], the European Cities [21], China [22], and the United States [23]. This method developed the aggregate SDG Index to evaluate the current state of sustainable development. Second, to measure the distance to the SDG targets, the Organization for Economic Co-operation and Development (OECD) developed a unique methodology across goals and targets since 2017 [24,25]. This method could assess trends over time and transboundary effects compared with the former method, but it does not result in a composite index.

In the present study, we not only tried to assess the trend of goals over time but also to assess the performances of coordination between every SDGs. We developed two multivariate indices based on SDG indicators, namely a development index and a coordination index. The former index can be used to access the degree (or speed) of sustainable development of a region whereas the latter index was designed to measure the coordination among the degrees of sustainable development accessed different categories of indicators. Such coordination can be interpreted as the quality of sustainable development. The core in developing these two indices lies in the entropy weight coefficient method [26,27], by which the weights of SDG indicators were objectively determined.

## 2. Study Area and the Strategy

### 2.1. Study Area Selection: Fujian Province, China

To help us select the study area, we proposed two principles. First, the study area should be representative or extraordinary. Assessing sustainable development means that we evaluated the degree of coordination among society, the economy, and the environment. We should select a study area that has better or worse development because the development in the study area was extraordinary. Second, the data should be accessible for a long time for the study area. Data were essential if we wanted to construct an index based on indicators. In addition, there should be a large quantity of data. We believe that high-quality data is defined as those that are high-precision, cover the full study area, and have a long time series.

According to the two principles, we selected the Fujian Province in China. First, Fujian Province is one of the Ecological Civilization Pilot Zones in China. The government of Fujian Province launched policies to implement sustainable development. Therefore, Fujian is representative. Secondly, since the Digital Fujian project was launched in 2000, Fujian Province has made great progress in statistical work. In particular, the geographic information industry has a high level of development, and the statistical data are open. Therefore, it was significant to assess the degree of sustainable development for Fujian Province.

### 2.2. Constructing SDGs Indicators-Based Indices: A Strategy

Constructing a composite index needs to base on composite indicators. Before we constructed an index, we needed to conduct some preparatory work on the indicators, data, and weights. The three steps for constructing indices based on indicators are as follows:

(1) Selecting indicators, collecting data, and processing data.
(2) Determining the weights of different indicators based on entropy.
(3) Calculating two multivariate indices based on SDGs indicators.

In the first step, we selected indicators by certain principles and collected data from certain materials. We also needed to process data according to certain criteria. In the second step, we applied the entropy weight coefficient method to determine the exact weights of the indicators. In the third step, we calculated the development index and coordination index to assess the sustainable development performance based on the weights and indicators.

## 3. Selecting Indicators, Collecting Data, and Processing Data

### 3.1. Selecting Indicators

Sustainable development is a complex system that contains many subsystems or dimensions. To simplify inherently complex relationships of this system, scholars often focus on subsystems or dimensions. Previous studies have expressed that indicators should be selected based on different dimensions [28]. For example, the United Nations Development Programme proposed the human development index to assess sustainability in three categories: income, longevity, and education [29]. Kaivo-oja, et al. [30] indicated that the framework of the sustainable society index included human well-being, environmental well-being, and economic well-being. To account for the correlation between land urbanization and population urbanization [31], Shen and Zhou [32] proposed sustainable urbanization indicators from four dimensions, namely, the economy, society, the environment, and governance. Zhou, et al. [33] introduced the framework of the responsibility-based method by using 20 responsibility departments. To better analyze sustainable development, scholars tried to divide 17 SDGs into multivariate dimensions. Sustainable development generally relates to social harmony, ecological friendliness, and economic development. The United Nations SDGs include 17 goals, and these goals cover all aspects of society, the economy, and the environment. The pursuits of these three dimensions and these 17 goals are the same. “Transforming our World: the 2030 Agenda for Sustainable Development” [34] presented the 5Ps, that is, People (Goal 1–5), Planet (Goal 6, 12–15), Prosperity (Goal 7–11), Peace (Goal 16) and Partnership (Goal 17). Fu, et al. [35] regarded SDGs as an attribute that is a product of society and divided the 17 SDGs into three categories, namely, essential needs (Goal 2, 6, 7, 14, and 15), expected objectives (Goal 1, 3, 4, 5, 8, 10, and 16), and governance (Goal 9, 11, 12, 13, and 17). According to the concept that environment support economic and social development, Stockholm Resilience Centre developed a model called “the wedding cake” [36]. This model divided the 17 SDGs into three levels, namely biosphere as the bottom level (Goal 6, 13, 14, 15), society as the middle level (Goal 1, 2, 3, 4, 5, 7, 11, and 16), and economy as top level (Goal 8, 9, 10, 12, and 17).

To explore the degree of regional sustainable development, we divided the indicator system into three dimensions by following the most popular classification [12,37,38], namely, the social, economic, and environmental dimensions. These three dimensions are widely referred to as the “three pillars” of sustainable development or “triple bottom line” [39]. To organize goals into different dimensions, we proposed that the social dimension should include the goals of dignity, health, equality, and security related to humans. The economic dimension should include the goals of prosperity and industry. The environmental dimension should include the goals of resource, creature, and climate. Therefore, the social dimension included Zero Hunger (Goal 2), Good Health and Well-being (Goal 3), Quality Education (Goal 4), Gender Equality (Goal 5), Sustainable Cities and Communities (Goal 11), Peace, Justice and Strong Institutions (Goal 16), and Partnerships for the Goal (Goal 17). The economic dimension included No Poverty (Goal 1), Decent Work and Economic Growth (Goal 8), Industry, Innovation and Infrastructure (Goal 9), Reduced Inequalities (Goal 10), and Consumption and Production (Goal 12). The environmental dimension included Clean Water and Sanitation (Goal 6), Affordable and Clean Energy (Goal 7), Climate Action (Goal 13), Life below Water (Goal 14), and Life on Land (Goal 15).

We proposed two principles to help us select indicators. First, we selected indicators from China’s SDGs indicators. China’s SDGs indicators were authoritative and comprehensive for the region of China [40], which launched localization sustainable development indicators in 2018. Second, the indicators should be accessible for a long time. Regardless of whether the type of indicator is a raster or observed data, it should be accessible for a long time. According to these principles, we selected about 59 indicators to belong to the 17 goals. First, according to China’s SDGs indicators, we selected 123 indicators. There are 163 China’s SDGs indicators, but 123 of the 163 indicators can be quantified. Second, according to the accessibility of the indicators over a long period, we selected 59 indicators from 123 China’s SDGs indicators. When we assessed regional sustainable development, we modified the indicators based on China SDGs indicators because China SDGs are only suitable for the national level. Finally, we selected 59 indicators, 15 categories, and three dimensions, as shown in Table 1.

### 3.2. Collecting Data

The period of data for all indicators was set from 2007 to 2017. One of the principles for selecting the indicators was the accessibility of the indicators for a long time period. According to this principle, the data were the most complete for the indicators from 2007 to 2017. The length of time conformed to the demands of the calculation.

The collected data come from different statistical materials. Specifically, “En15-2” come from the Statistical Yearbook of China. “En6-3” and “En6-4” come from the Water Resources Bulletin of Fujian Province. “E1-1” and “E1-2” come from the Poverty Monitoring Report of Rural China. “En15-1” comes from the Statistical Bulletin of Fujian Province. The other indicators come from the Statistical Yearbook of Fujian Province.

The indicator system for Fujian sustainable development has 15 of the 17 SDGs, excluding Goal 5 (Gender Equality) and Goal 14 (Life below Water). This is not to say that these goals were not related to the sustainable development of Fujian; rather, the suitable indicator selection was impacted by data limitations.

### 3.3. Processing Data

We performed three processes for data based on the China SDGs indicators.

- First, we converted the ratio of the change to observed values, such as “GDP per capita growth rate” to “GDP per capita”, and “GDP growth rate” to “GDP”. The types of China SDGs indicators included observed values, ratios, and ratios of change. According to the theory of the entropy method, it will impact the weight of the indicator if the indicator is a ratio of change.
- Second, according to the data accessibility, we modified indicators based on China SDGs indicators. For example, “fertilizer consumption” replaced “ratio of fertilizer consumption” in Goal 2. “Death due to road traffic injuries” replaced “death ratio due to road traffic injuries” in Goal 3. The “Engel coefficient” and “urban-rural income distribution” replaced the “Gini coefficient” in Goal 10. The “contribution ratio of the tertiary industry to the GDP” replaced the “contribution ratio of tourism to the GDP” in Goal 12. “Civil litigation” and “Administrative litigation” replaced “Crime rate” in Goal 16.
- Third, the value of the “En7-1” indicator in 2017 was not consistent with those of other years. We adjusted it to be the same as in 2016. According to the concept of entropy method, the value of each indicator must be a positive number or negative number. If an indicator contains a positive number and a negative number, the weights of the indicator are invalid.

The effectiveness of indicators may impact the value of the indices. To guide sustainable development, the indicators must be effective. First, if the type of indicator data is the ratio of change, e.g., GDP per capita growth rate, GDP growth rate, and population growth rate, the weight of the indicator may be different with the use of the entropy weight coefficient method. The weight of an indicator was invalid if the value of the ratio of change for an indicator was 0 from 2007 to 2017. On the contrary, if the value of an indicator was 100 from 2007 to 2017, the ratio of change for the indicator was 0 and the weight of the indicator is valid. Secondly, the results of a sustainable development index may have been different with the use of the entropy weight coefficient method if we only selected the observed values of indicators. In addition, the indicators should cover each dimension of sustainable development, and the number of indicators under each dimension should not be too small or large.

## 4. Determining the Weights of Different Indicators Based on Entropy

We needed to calculate the weights of the indicators by using a particular method before we developed an index. To assess the spatio-temporal progress of sustainable development, systematic methods are vital, and the weights of indicators are a problem that each method must solve. The current body of literature has indicated that there are many methods for calculating the weights. Equal weight [41] is the simplest and most convenient method, but the results are not objective. To calculate the objective weights, scholars have developed the principal component analysis [42] and TOPSIS [43]. The entropy weight coefficient method is also one of the major methods (e.g., [44,45,46,47]). 

To determine the weights of different indicators, we selected the entropy weight coefficient method for three reasons. First, stakeholders may be unwilling to obtain equal weight or have using preference. Second, subjective weight can lead to error caused by the bias of decision-makers. Third, the entropy weight coefficient method has been widely recognized because it calculates the weights of indicators based on the data set itself and the calculation is easier to operate. Entropy can explain information or uncertainty in the field of information theory [48,49,50,51]. It has wide applications, such as water quality assessment, engineering, and economy [52]. The development of the entropy weight coefficient method has extended to regional development [27]. Current studies have validated that the entropy weight coefficient method can be used to calculate the weights of a group of indicators and assess the performance of development [44].

There are four steps for applying the entropy weight coefficient method [26,27,53]. It is assumed in this paper, that *i* represents the indicator, namely, *i*=1, 2, 3… n; *j* represents the sample, namely, *j*=1, 2, 3… m; and $x_{ij}$ represents the original value of the *i*th indicator and the *j*th sample. First, to eliminate the magnitudes of the indicators, it was necessary to normalize the indicators. According to the attribution of the indicators, we divided them into positive and negative effects.

When the indicator had positive effects, the following equation was used:

(1) $$R_{ij}=\frac{x_{ij}}{max_{j}\left(x_{ij}\right)}$$

When the indicator had negative effects, the following equation was used:

(2) $$R_{ij}=\frac{min_{j}\left(x_{ij}\right)}{x_{ij}}$$

where $R_{ij}$ represents the normalized value of $x_{ij}$. $max_{j}\left(x_{ij}\right)$ and $min_{j}\left(x_{ij}\right)$ show the largest and smallest values among all $x_{ij}$. Second, after normalization, it was necessary to calculate the proportion of $R_{ij}$, divided by the sum of $R_{ij}$, as follows:

(3) $$f_{ij}=\frac{R_{ij}}{\sum_{j}^{m}R_{ij}}$$

Third, according to the value of $f_{ij}$, the formula of the entropy value was written as follows:

(4) $$h_{i}=-k\overset{m}{\underset{j=1}{\sum }}f_{ij}\times \ln f_{ij}$$

(5) $$k=\frac{1}{\ln m}$$

where $h_{i}$ represents the value of the entropy of each indicator. Fourth, according to the value of entropy among the indicators, we needed to determine the weights. The weights of different indicators were calculated as follows:

(6) $$w_{i}=\frac{1-h_{i}}{\sum_{i=1}^{n}\left(1-h_{i}\right)}$$

where $w_{i}$. represents the weight of each indicator.

## 5. Calculating Two Multivariate Indices Based on SDGs Indicators

To assess the degree of sustainable development, we calculated two multivariate indices based on the weight of indicators, namely, the development index and the coordination index. For the process of regional sustainable development, the two multivariate indices can explain the speed and quality of development. In other words, the development index assesses the speed of sustainable development. The coordination index represents the quality of coordination in the development among society, economy, and environment. The calculation formulas are in the following sections.

### 5.1. Development Index

According to the weights of the indicators in Equation (6) and the standardized values in Equation (3), we calculated the development index. The development index meant that we used the weighted sum method between the weights and standardized values. The formula for calculating the development index was as follows [27,53]:

(7) $$E_{j}=\overset{n}{\underset{i=1}{\sum }}w_{i}\times R_{ij}$$

where $E_{j}$ represents the development index of sample *j*. According to the indicator system in Table 1, the indicators were divided into three dimensions: social, economic, and environmental. Following Equation (7), we calculated the performance of the development index for the different dimensions.

The development index of the social dimension is as follows:

(8) $$E_{j\left(s\right)}=\overset{n_{s}}{\underset{i=1}{\sum }}w_{i\left(s\right)}\times R_{ij\left(s\right)}$$

The development index of the economic dimension is as follows:

(9) $$E_{j\left(e\right)}=\overset{n_{e}}{\underset{i=1}{\sum }}w_{i\left(e\right)}\times R_{ij\left(e\right)}$$

The development index of the environmental dimension is as follows:

(10) $$E_{j\left(en\right)}=\overset{n_{en}}{\underset{i=1}{\sum }}w_{i\left(en\right)}\times R_{ij\left(en\right)}$$

where $E_{j\left(e\right)}$, $E_{j\left(s\right)}$, and $E_{j\left(en\right)}$ represents the development index of three dimensions, namely, the social, economic, and environmental dimensions, respectively. $n_{e}$, $n_{s}$, and $n_{en}$ represent the number of indicators for the three dimensions.

To assess the degree (speed) of sustainable development, we classified the values of the development index into three levels according to previous studies [27,54], namely strong, medium, and weak, as shown in Table 2.

### 5.2. Coordination Index

The coordination index assesses the quality of sustainable development by considering the coordination among society, economy, and environment. The formula for calculating the coordination index was as follows and was based on the development index of the three dimensions [27,53]:

(11) $$G_{j}=1-\frac{S_{j}}{\bar{E}}$$

(12) Sj=13[(Ej(s)−E¯)2+(Ej(e)−E¯)2+(Ej(en)−E¯)2]

(13) E¯=(Ej(e)+Ej(s)+Ej(en))3

where $G_{j}$ represents the coordination index of sample *j*. $S_{j}$ represents the standard deviation of the development index for three dimensions. $\bar{E}$ represents the arithmetic mean of the development index for the three dimensions.

To assess the degree (quality) of coordination in the development, we classified the coordination index. The degree of the sustainable coordination index could be classified into three levels, namely, strong, medium, and weak [27,54], as shown in Table 3.

## 6. Results and Analysis

### 6.1. Results

According to Section 5 and Section 6, we calculated the weights of the indicators, development index, and coordination index (Table 4). Table 4 also includes the development index of social, economic, and environmental dimensions.

The sustainable development index based on the indicators could also be used to calculate the development index of six categories in the social dimension, that is, Goal 2, Goal 3, Goal 4, Goal 11, Goal 16, and Goal 17 (Table 5).

### 6.2. Analysis

To analyze the results of Section 6.1, we used the McKinsey Matrix method, which was developed based on the Boston Consulting Group matrix and was modified by McKinsey [55]. This method can help stakeholders or companies to develop marketing strategies. The McKinsey Matrix assesses the performance in the competitiveness and attractiveness dimensions of a company [56]. According to the levels of the two dimensions, the marketing strategies of the company were divided into three categories: high, medium, and low [57]. Similar applications of the McKinsey Matrix method can be found in Shen et al. [27].

**Figure 1:**
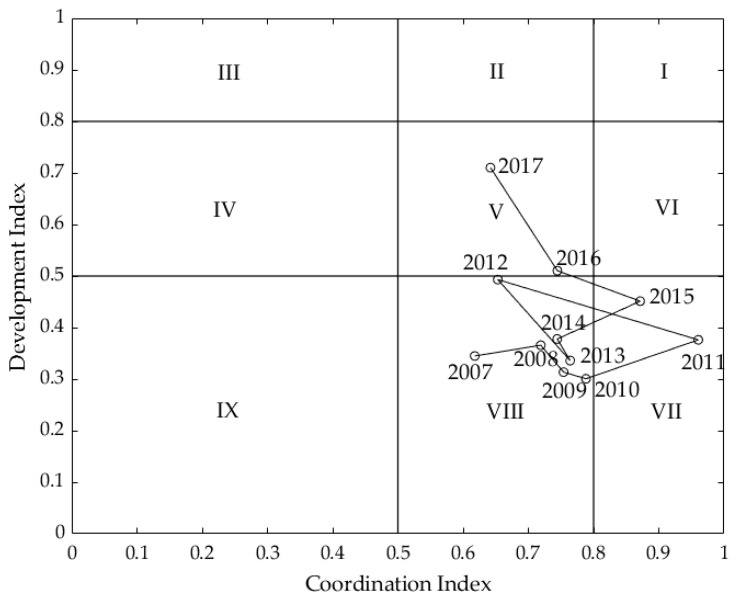
The matrix of sustainable development from 2007 to 2017, Fujian Province.

Using the McKinsey Matrix method, we visualized the results in Section 6.1, as shown in Figure 1. It can be seen in this figure that in 2017, the performance of sustainable development was in Area V. This result indicates that Fujian had a medium development speed and medium development quality. With the development of economic globalization, the development speed of Fujian has improved. The government of China asked that local governments must take measures to protect the environment by proposing the Ecological Civilization. Fujian Province is trying to positively improve the living environment and coordinate between social, economic, and environmental development. To achieve the level of strong development according to the development index and coordination index, we proposed the following: (1) Fujian should improve the level of urbanization and (2) Fujian should increase the proportion of high-tech industries and tourism.

In terms of the whole period, the changing trends of the development index and coordination index were different in Fujian Province from 2007 to 2017. Specifically, the development index changed from showing weak development (Area VIII in Figure 1) to showing medium development (Area V), demonstrating that the development speed of Fujian has improved. By contrast, the coordination index changed from medium coordination (Area VIII) to strong coordination (Area VII) from 2007-2011, showed medium coordination (Area VIII) from 2012-2014, and finally changed from strong coordination (Area VII) to medium coordination (Area V) from 2015-2017. Such a trend shows that the degree of coordination among society, the economy, and the environment was fluctuating. It is also noticed that the changing direction of the coordination index shifted in 2011 and 2015.

**Figure 2:**
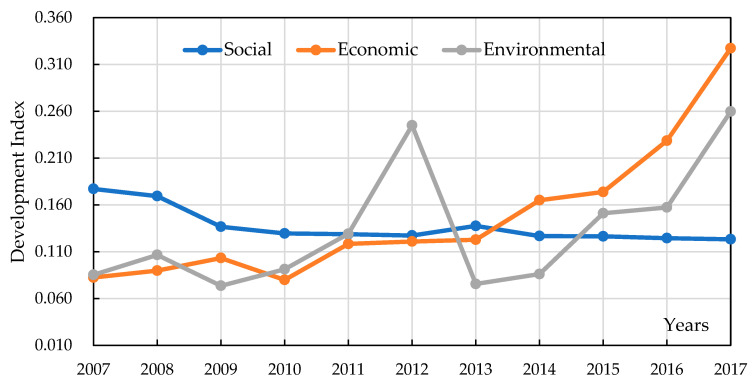
The trend of the development index for three dimensions from 2007 to 2017.

To explain this shift, we further analyzed the related development. According to the values of the development index for the social, economic, and environmental dimensions, we showed the changing trends of the development index for three dimensions from 2007 to 2017, as shown in Figure 2. It can be found in this figure that before 2012, the trend of the three dimensions became synchronized. This was the main reason why the coordination index had an obvious variation from 2011 to 2012. In 2012, the value of the environmental development index suddenly improved 2016, the value of the social development index was lower than those of the economic and environmental dimensions. The trend of the three dimensions became synchronized in 2014 and 2015. This was the main reason why the coordination index changed from strong coordination to medium coordination in 2015 and 2016.

It is also noted in Figure 2 that that the social performance of Fujian fell slightly in the past 11 years. Simultaneously, the development index values for the economic and environmental dimensions greatly increased. On the one hand, we proposed that the social dimension was the main reason why the coordination index of Fujian showed medium coordination in 2017. Therefore, we recommend that the government of Fujian should improve the performance of the social dimension in the process of sustainable development. On the other hand, we cannot explain why the development index of the social dimension was declining.

**Figure 3:**
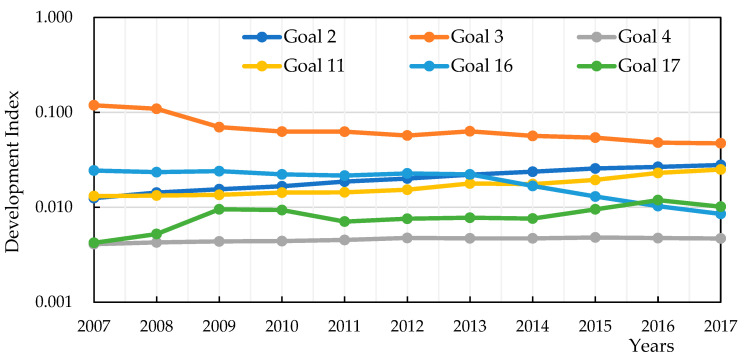
The trend of the development index for six social goals from 2007 to 2017.

To explain the reason, we further analyzed the development index of six goals for the social dimension. The social dimension includes six goals, namely, Goal 2 (s2), Goal 3 (s3), Goal 4 (s4), Goal 11 (s11), Goal 16 (s16), and Goal 17 (s17). There were 31 indicators in the six goals. We showed the changing trends of the development index for these six goals, as shown in Figure 3. It can be seen in Figure 3 that the changing trends of Goal 2, Goal 4, Goal 11, and Goal 17 slightly improved from 2007 to 2017. On the contrary, the changing trends of Goal 3 and Goal 16 declined. During the past 11 years, the development index values of Goal 3 (Good Health and Well-being) and Goal 16 (Peace, Justice, and Strong Institutions) declined. Thus, the performance on Goals 3 and 16 might be the main reason why the values of the development index for the social dimension decreased. In addition, the indicators related to Goal 3 and Goal 16 include “death due to road traffic injuries”, “health workers density”, “prevalence”, “civil proceedings”, and “administrative litigation”. Fujian has implemented measures to improve these indicators, but this was not a significant change. In other words, Fujian needed to input more resources to improve social services and social security.

## 7. Conclusions

To assess the degree of sustainable development, it is vital to construct a composite index based on comprehensive indicators. Much efforts have been made towards this end [58,59,60,61]. In this study, we constructed composite indices based on the SDGs (Sustainable Development Goals) established by the United Nations. We selected a study area with two principles. Based on the study area, we selected the localization of indicators by two principles. We collected and processed indicator data from 2007 to 2017. We constructed two multivariate indices based on data by applying the entropy weight coefficient method. According to the results, we can draw three major conclusions:

(1) In 2017, the sustainable development of Fujian showed medium development and medium coordination. From 2007 to 2017, the development speed improved from weak development to medium development. The changing trends in the development index improved. The coordination index changed from medium coordination to strong coordination before 2011, then changed to medium coordination from 2012 to 2014, and finally, changed from strong coordination to medium coordination from 2015-2017. The changing trends of the coordination index fluctuated.

(2) The main reason why the coordination index of Fujian decreased in 2012 was that the value of the environmental development index suddenly improved. The main reason why the coordination index of Fujian decreased in 2016 was that the value of the social development index decreased.

(3) The main reason why the coordination index of Fujian showed medium coordination was that the development index of Goal 3 (Good Health and Well-being) and Goal 16 (Peace, Justice, and Strong Institutions) was low. The decision-makers of Fujian should take measures to improve well-being and social security.

One limitation of this study lies in the classification of indicators. Here, we first classified the SDGs into three dimensions (society, economy, and environment) and then employed different sets of indicators to measure each SDG. However, the sets of indicators can be improved and in some cases, one indicator can be used for multiple SDGs.

It is also important to note that the entropy employed in this study is essentially Shannon entropy, also called information entropy. Recently, Boltzmann entropy (or thermodynamic entropy, configurational entropy) has been revisited [62,63], and several computational methods have been developed [64,65,66,67,68,69,70]. According to the father of contemporary ecological thermodynamics [62], Boltzmann entropy is actually more suitable to be applied in landscape ecology to explore the thermodynamic interpretations of landscape dynamics. In the future, we hope to apply Boltzmann entropy to develop sustainability indices and explore the difference in performance between such indices and the two used in this study. It is also our hope that our results are of use to stakeholders (government, companies, and non-profit organizations) in promoting sustainable development.

## Figures and Tables

**Table 1:** Indicator system created by selecting for sustainable development.

Dimension	Goal	Indicator	Attribution
Social	Goal 2	S2-1 Prevalence of undernourishment	−
		S2-2 Cereal yield per capita	+
		S2-3 Per capita disposable income of rural residents	+
		S2-4 Fertilizer consumption	−
		S2-5 Arable land area per capita	+
		S2-6 Agriculture, forestry and water expenditure (% finance expenditure)	+
		S2-7 Price index of household consumption in food (last=100)	−
	Goal 3	S3-1 Death due to road traffic injuries	−
		S3-2 HIV prevalence	−
		S3-3 Tuberculosis prevalence	−
		S3-4 Malaria prevalence	−
		S3-5 Number of traffic accidents (per 10,000)	−
		S3-6 Health workers (per 10,000)	+
		S3-7 Number of beds in medical institutions (per 10,000)	+
	Goal 4	S4-1 Early education (%)	+
		S4-2 Number of college students (per 10,000)	+
		S4-3 Number of high school students (per 10,000)	+
		S4-4 Number of high school teachers (per 10,000)	+
		S4-5 Number of college teachers (per 10,000)	+
		S4-6 Proportion of educational funds expenditure in GDP	+
	Goal 11	S11-1 Railway mileage per capita	+
		S11-2 Highway mileage per capita	+
		S11-3 Urban-rural and community expenditure (% finance expenditure)	+
		S11-4 Urban green areas per inhabitant	+
		S11-5 Percentage of living waste processed	+
		S11-6 Recycling rate of industrial solid waste	+
	Goal 16	S16-1 Civil litigation	−
		S16-2 Administrative litigation	−
	Goal 17	S17-1 Local finance revenue (% GDP)	+
		S17-2 Local tax revenue (% finance revenue)	+
		S17-3 Energy conservation and environment protection expenditures (% finance expenditure)	+
Economic	Goal 1	E1-1 Proportion of population living below the national poverty line	−
		E1-2 Poverty rates	−
		E1-3 Urban residents protected by the minimum standard of living (% urban population)	−
		E1-4 Rural residents protected by the minimum standard of living (% rural population)	−
		E1-5 Disabled people protected by the minimum standard of living (% total population)	−
		E1-6 People affected by natural disasters	−
	Goal 8	E8-1 GDP per capita	+
		E8-2 GDP	+
		E8-3 Labor productivity	+
		E8-4 Urban unemployment rate	−
	Goal 9	E9-1 Volume in passenger transport	+
		E9-2 Volume in freight transport	+
		E9-3 Proportion of R&D expenditure in the GDP	+
	Goal 10	E10-1 Urban Engel coefficient	−
		E10-2 Rural Engel coefficient	−
		E10-3 Urban-rural income distribution	−
	Goal 12	E12-1 Contribution ratio of the tertiary industry to the GDP	+
Environmental	Goal 6	En6-1 Percentage of population with safe and adequate drinking water in urban areas	+
		En6-2 Proportion of rural households with sanitary toilets	+
		En6-3 Proportion of surface water quality reaching or better than Class III water	+
		En6-4 Proportion of surface water quality worse Class V water	−
		En6-5 Urban anthropogenic wastewater that receives treatment (%)	+
		En6-6 Water resources per capita	+
	Goal 7	En7-1 Reduced energy consumption per unit of GDP	+
	Goal 13	En13-1 Deaths due to natural disasters (per 100,000)	−
		En13-2 Total damages attributed to disasters as % of GDP	−
	Goal 15	En15-1 Forest area as a proportion of the total land area	+
		En15-2 Proportion of protected and conserved terrestrial areas	+

**Table 2:** The classification of the development index (*E*).

Development Index	The Level of Development
0.8 ≤ *E* ≤ 1	Strong development
0.5≤ *E* < 0.8	Medium development
0 ≤ *E* < 0.5	Weak development

**Table 3:** The classification of coordination index (G).

Coordination Index	The Level of Coordination
0.8 ≤ *G* ≤ 1	Strong coordination
0.5 ≤ *G* < 0.8	Medium coordination
0 ≤ *G* < 0.5	Weak coordination

**Table 4:** Results from the sustainable development index.

Years	Social		Economic		Environmental		*E_j_*	*S_j_*	$\bar{E}$	*G_j_*
	*E_j(s)_*	*w_i_*	*E_j(e)_*	*w_i_*	*E_j(en)_*	*w_i_*				
2007	0.177	0.241	0.082	0.329	0.085	0.430	0.345	0.044	0.115	0.618
2008	0.169		0.090		0.107		0.366	0.034	0.122	0.719
2009	0.137		0.103		0.074		0.314	0.026	0.105	0.754
2010	0.130		0.080		0.091		0.301	0.021	0.100	0.788
2011	0.129		0.118		0.129		0.376	0.005	0.125	0.960
2012	0.127		0.121		0.245		0.493	0.057	0.164	0.653
2013	0.138		0.123		0.076		0.336	0.026	0.112	0.764
2014	0.127		0.165		0.086		0.378	0.032	0.126	0.744
2015	0.126		0.174		0.151		0.451	0.019	0.150	0.871
2016	0.124		0.229		0.157		0.510	0.043	0.170	0.745
2017	0.123		0.327		0.260		0.710	0.085	0.237	0.642

Note: $E_{j}$ represents the development index. *w_i_* represents weights. $G_{j}$ represents the coordination index. $S_{j}$ represents the standard deviation of the development index for three dimensions (i.e., *E_j(s),_ E_j(e),_* and *E_j(en)_*). $\bar{E}$ represents the arithmetic mean of the development index for the three dimensions.

**Table 5:** Results on the development index of six social categories (*E_j(s2),_ E_j(s3),_ E_j(s4),_ E_j(s11),_ E_j(s16),_* and *E_j(s17)_*).

Years	Development Index					
	*E_j(s2)_*	*E_j(s3)_*	*E_j(s4)_*	*E_j(s11)_*	*E_j(s16)_*	*E_j(s17)_*
2007	0.012	0.119	0.004	0.013	0.024	0.004
2008	0.014	0.109	0.004	0.013	0.023	0.005
2009	0.015	0.070	0.004	0.014	0.024	0.010
2010	0.017	0.063	0.004	0.014	0.022	0.009
2011	0.019	0.063	0.005	0.014	0.022	0.007
2012	0.020	0.057	0.005	0.015	0.023	0.008
2013	0.022	0.063	0.005	0.018	0.022	0.008
2014	0.024	0.056	0.005	0.018	0.017	0.008
2015	0.026	0.054	0.005	0.019	0.013	0.010
2016	0.027	0.048	0.005	0.023	0.010	0.012
2017	0.028	0.047	0.005	0.025	0.009	0.010
